# Basking sharks overlap with primary and secondary deep scattering layers during overwintering migration in the Northwest Atlantic Ocean

**DOI:** 10.1371/journal.pone.0348589

**Published:** 2026-06-03

**Authors:** Jaida N. Elcock, Martin C. Arostegui, Laura H. McDonnell, C. Antonia Klöcker, Gregory B. Skomal, Simon R. Thorrold, Camrin D. Braun

**Affiliations:** 1 Biological Oceanography, Massachusetts Institute of Technology-Woods Hole Oceanographic Institution Joint Program in Oceanography/Applied Ocean Science and Engineering, Cambridge, Massachusetts, United States of America; 2 Biology Department, Woods Hole Oceanographic Institution, Woods Hole, Massachusetts, United States of America; 3 School of Applied Sciences, Edinburgh Napier University, Sighthill Campus,‌‌ Edinburgh, United Kingdom; 4 Massachusetts Division of Marine Fisheries, New Bedford,‌‌ Massachusetts, United States of America; University of Messina, ITALY

## Abstract

Long-distance migrations allow animals to exploit seasonal prey opportunities and track favorable oceanographic conditions. The basking shark (*Cetorhinus maximus*) is a large, filter-feeding elasmobranch commonly observed in temperate shelf habitats, though it is known to seasonally occupy warmer, lower-latitude regions. In the Northwest Atlantic Ocean, basking sharks migrate from summer habitats on the continental shelf of the northeastern United States and Canada to the tropical waters of the Caribbean and South America during winter. However, the functional role of these large-scale movements is poorly understood, and their overwintering behavior during migration remains enigmatic. Here, we use pop-up satellite archival transmitting (PSAT) tags to measure basking shark vertical habitat use during this migration. Based on daily summaries of time-at-depth and time-at-temperature, we find that sharks exhibited two main behaviors: shallow epipelagic occupancy on or near the continental shelf and movements throughout the mesopelagic in offshore waters. While offshore, vertical habitat use was characterized by a strong diel vertical migration (DVM) that overlapped with primary and secondary deep scattering layers, particularly in the southern Sargasso Sea. However, DVM behavior was widespread throughout the Sargasso Sea, where most tagged individuals overwintered, and continued into the Caribbean and during trans-equatorial movements. Our results suggest basking sharks likely forage throughout these large-scale migrations, rather than relying primarily on energy stores as has been suggested for other highly migratory shark species. We also suggest that basking sharks may regularly target prey biomass in a deeper, often non-migratory prey layer below the primary deep scattering layer. These findings highlight the potential ecological importance of mesopelagic prey for basking sharks during migration and contribute to growing recognition of the ecosystem services supported by deep-pelagic food webs within and beyond the primary deep scattering layer.

## Introduction

Many species undertake large-scale seasonal movements that track changes in productivity and prey distribution [1]. In the marine environment, such movements often link seasonal productivity hotspots [2], with migrants sometimes relying on energy gained to sustain long-distance travel through more oligotrophic habitats (e.g., white sharks; [3]). The Northwest Atlantic Ocean (NWA) is characterized by strong seasonal pulses of primary and secondary production that attract a diverse assemblage of highly migratory species, including endangered North Atlantic right whales and other baleen whales [4,5], wide-ranging seabirds [6], multiple elasmobranch species [7–10], and obligate mesopelagic mesopredators that rarely occupy surface waters [11]. Many of these migrations coincide with seasonal peaks in prey availability, underscoring the ecological importance of the NWA as a foraging ground that fuels, and potentially motivates, energetically costly long-distance movements.

Vertically structured prey layers are a common and prominent feature in the pelagic environment. These “deep scattering layers” (DSLs), first identified using active acoustics [12], represent aggregations of mesopelagic organisms, including zooplankton, crustaceans, small fishes, and squid [13–15]. Recent estimates suggest that biomass within these layers ranges from 1 to 20 Gt [14,16]. Hydroacoustic surveys, often combined with stratified net sampling, have demonstrated that these scattering layers can be broadly classified by depth, vertical migration behavior, and dominant faunal groups, even when fine-scale taxonomic resolution is challenging (e.g., [17,18]). In many regions, vertically structured scattering layers include a primary diel-migrating layer in the upper to middle mesopelagic and, in some cases, deeper and more weakly migrating or non-migratory layers [15,17]. Organisms within these layers often migrate toward the surface at night in a process known as diel vertical migration (DVM, [14,15,18]), which plays an important role in linking surface productivity with deeper ocean ecosystems [19]. Predators frequently track this movement, diving to exploit vertically structured prey fields [20]. However, the extent to which these deep prey layers structure large-scale migratory movements of predators remains poorly understood.

Basking sharks (*Cetorhinus maximus*) are the world’s second largest fish (max size 12 m, [21]) and are known to make extensive, large-scale migrations from cooler, temperate waters into warmer (sub)tropical waters, including documented trans-Atlantic [22,23] and trans-equatorial movements [24]. While basking sharks in the Northeast Atlantic undertake seasonal southerly migrations spanning up to ~20º of latitude [25], individuals tracked in the NWA exhibit much more extensive winter movements. Many travel >50º of latitude and >17,000 km intra-annually and frequently return the following summer to the Northeast United States shelf, indicating strong site fidelity [24,26]. These movements occur in oligotrophic regions during which the sharks primarily occupy mesopelagic waters (~80% of time spent at depths of 200−1,000m [24,26]). Low productivity in surface waters during much of this migration suggests that epipelagic foraging opportunities may be limited. Biomass within mesopelagic communities may, therefore, serve as a critical food resource to support these migrations, a pattern documented in other pelagic predators (e.g., albacore [27], oceanic whitetip sharks [28], and tope/soupfin sharks [29]) and the topic of several recent meta-analyses [30,31] and reviews (e.g., [20,32]).

Many studies suggest that basking shark movements are used to exploit seasonal hotspots of zooplankton productivity [21,26,33,34] as this species has been shown to effectively respond to fine-scale variability in zooplankton density [35–37]. Previous studies have proposed that southward movements of Atlantic basking sharks are driven by seasonal declines in copepod abundance at higher latitudes, coupled with access to warmer and potentially more productive waters during winter months [24–26]. However, the function of prolonged occupation of deep, pelagic waters far from continental shelves during these seasonal migrations remains unknown. Here, we use a satellite archival tag dataset to investigate basking shark behavior during their seasonal migratory cycle. We hypothesize that basking sharks frequent the deep ocean in oligotrophic waters to overlap with high concentrations of mesopelagic biomass and that foraging at depth in these scattering layers supports their large-scale horizontal movements.

## Methods

Basking sharks were opportunistically tagged with three different types of pop-up satellite archival transmitting (PSAT) tags (Models Mk10-PAT, Mk10-AF, miniPAT; Wildlife Computers, Inc., WA, USA) near the coast of Cape Cod, Massachusetts, in the NWA between 2004 and 2011 (n = 57 individuals). Complete details of the tagging methodology can be found in Braun et al. (25). Tagging procedures were approved by the Institutional Animal Care and Use Committee (IACUC) at the Woods Hole Oceanographic Institution following protocol #16518. No additional permitting was required. Briefly, PSAT tags recorded depth, temperature, and light-level data (3–30 seconds sampling interval, depending on tag model and year) to onboard memory that can be extracted if the tag is physically recovered. Tags that were not recovered reported several summarized data products via satellite, including a time series of depth and temperature at temporal resolutions ranging from 75 seconds to 10 minutes, as well as time-at-depth (TAD) and time-at-temperature (TAT) data summarized at daily or sub-daily timescales. These transmitted TAD and TAT data from each tag were compiled into 24-hour summaries of the percentage of time spent in each depth bin and standardized to shared depth bins (0-10m, 10-25m, 25-50m, 50-200m, 200-400m, 400-1000m, and 1000-2000m) and shared temperature bins (0–7ºC, 7–9ºC, 9–11ºC, 11–13ºC, 13–15ºC, 15–17ºC, 17–19ºC, 19–21ºC, 21–23ºC, 23–25ºC, and >25ºC) across tag models and deployment years. We adjusted times to local time for all subsequent analyses, using the daily location estimates (see below). All analyses were conducted in the R Statistical Environment [38].

### Transmitted daily summary data

To characterize large-scale patterns of vertical habitat use across individuals and deployments, we analyzed satellite-transmitted daily summaries of time-at-depth (TAD) and time-at-temperature (TAT). For clustering, we used an arcsine transformation on the TAD and TAT proportions to improve normality of these skewed data. We created a distance matrix to assess the similarities of each day of TAD and TAT data based on the seven common depth bins and 11 common temperature bins. We computed pairwise distances among samples using the Manhattan (“city-block”) metric, which sums absolute differences across dimensions, rather than Euclidean distance as the latter is known to be sensitive to differences in the scale and units of variables; therefore, standardization is generally required when attribute scales differ substantially [39]. In contrast, Manhattan-type metrics are widely used for quantitative variables measured on discrete or binned scales (e.g., counts, depth bins). From the distance matrix, we created a cluster tree using the true Ward clustering method [40] because of its robustness when handling outliers. We assessed the optimal number of clusters based on our data using the NbClust package for R [41].

For each day represented by TAD and TAT data, a geographic position (latitude and longitude) was estimated using a state-space hidden Markov model from the HMMoce package [42] for R. Further description of this methodology can be found in [26]. These positions were grouped by cluster to explore the geographic distribution of the resulting vertical behavior patterns. Days assigned to a given cluster were also identified within the available transmitted (n = 14) and archived (n = 2) time series data from 16 tags to investigate the fine-scale vertical habitat use within each cluster. All time series data were artificially coarsened to a common resolution (600 seconds) to standardize for visualization. The depth-temperature time series data were used to identify the onset of the southerly migration for each individual by calculating the date when the shark crossed the Gulf Stream North Wall, traditionally defined as where the 15ºC isotherm occurs at 200 m depth [43].

### Recovered high-resolution archival data

Archival data from the two recovered tags provided a unique opportunity to characterize vertical habitat use and behaviors at a higher resolution. These two tags recorded depth (pressure), temperature, and light-level data at either 3- or 30- second resolution (hereafter “Shark 1” and “Shark 2”, respectively). These data were used to 1) identify the periodicity and prevalence of diel patterns in depth (i.e., diel vertical migration [DVM] on ~24 hour period); 2) estimate depths of specific daytime isolumes; and 3) detect bioluminescence at depth as a proxy for potential prey distribution. These metrics were used by Klöcker et al. [44] to study basking shark behavior in the Northeast Atlantic, enabling comparison between regions.

We used a continuous wavelet analysis to evaluate the periodicity and presence of diel patterns in the high-resolution, depth-time series from the recovered tags (as described in [44]). Analyses were conducted with a Morlet wavelet (*x*_*0*_ = 6) using the *WaveletComp* package for R [45]. In addition to the default workflow, which applies global standardization of the time series, we implemented a local variance normalization using a sliding-window (7-day) z-score standardization prior to the wavelet transformation. This procedure accounts for non-stationary variance observed in the depth-time series (e.g., associated with habitat transitions), thereby allowing consistent assessment of periodicity. We assessed the statistical significance of the wavelet spectrum by generating 1,000 simulated time series for each individual based on a first-order autoregressive process (AR [1]) with p = 0.7 and using the observed mean. Wavelet power values that exceeded the bootstrapped 95% confidence limits were considered statistically significant and interpreted as evidence of non-random vertical migratory behavior within the time series. DVM was inferred when the wavelet power values at the 24-h period were statistically significant (p < 0.05). Although an ideal sinusoidal depth cycle would produce a single 24-h peak, real depth trajectories often include sharp descents or ascents or seasonal shifts in daylight regime, which generate additional harmonics (e.g., 12 h, 6 h) with diminishing power at higher frequencies. Because these harmonics do not reflect interpretable ecological behavior, they were excluded from further consideration.

To characterize habitat-specific differences in light penetration and identify depths likely favored by light-sensitive prey, we estimated the daily depth of a representative isolume for each individual using tag-recorded light measurements during daytime conditions (sun angle α > 6°). Only days with at least 20 valid observations were retained, and a three-day moving average was applied to smooth the resulting isolume depths for visualization. We focused on light-level values near 30 (LL30), corresponding to an irradiance range (~10^-10^-10^-11^ W cm^2^) commonly associated with preferred light environments of mesopelagic crustaceans (including known copepod prey in the North Atlantic) and global deep scattering layer communities [46]. Although the occupied light level range of such species may be broader, LL30 served as an approximate indicator of the depth at which vertically migrating zooplankton may aggregate. In addition, Shark 2 did not consistently experience light levels around LL30, so we also calculated LL40 and LL50 for both tags. LL40 and LL50 are one half and one order of magnitude brighter than LL30, respectively. Previous studies have shown that prey scattering layers, including the primary DSL, can locally align with light levels several orders of magnitude above the reported photobehavioral sensitivity of mesopelagic crustaceans [47–49]. Because the Wildlife Computers light sensor records relative light in a narrow blue wavelength band (415–460 nm) on a logarithmic scale [50], light-level values cannot be converted directly into absolute irradiance, and readings are not comparable between tags.

In addition to isolume depth, we used high-resolution, light-level time series to detect potential bioluminescence events as an *in situ* proxy for the presence of mesopelagic organisms. Following a modified version of the approach of Braun et al. [31], we applied a series of filters to identify sharp, short-lived increases in light level consistent with bioluminescent flashes rather than ambient light fluctuations. Events were discarded if they occurred in waters at least two orders of magnitude brighter than the tag light sensor’s sensitivity floor, or if the tag had just moved into shallower, brighter water (i.e., > 1 m ascent between time steps). Candidate flashes required an increase of more than two light-level units relative to the preceding two measurements and a peak value that exceeded expected noise based on the following two time steps and on the local standard deviation of the time series (calculated from 15–5 time steps prior). Because these filters eliminate periods where detection is unreliable, the method yields presence-only rather than quantitative estimates. All putative bioluminescence detections were visually checked against previously described flash characteristics for this light sensor to confirm or reject their classification. Because of these conservative quality-control measures, only the light-level data from the 3-second archival dataset was used to detect bioluminescent events.

## Results

**Fig 1:**
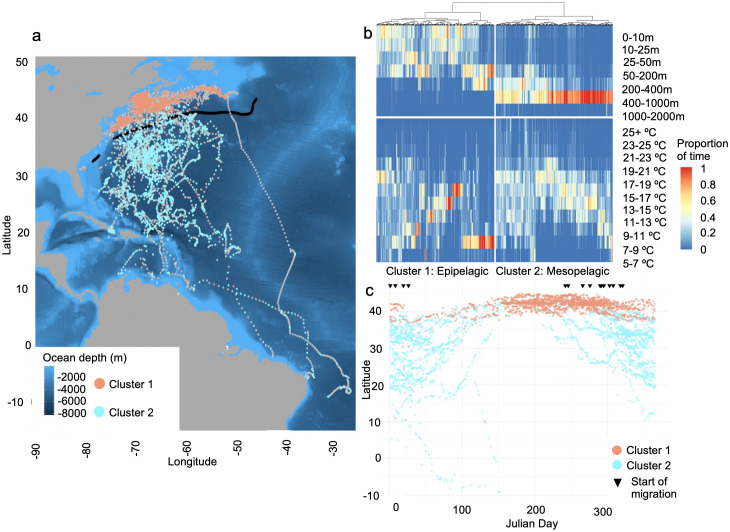
Basking shark TAT-TAD clusters. **A)** Basking shark movements throughout the Northwest Atlantic Ocean, B) assigned to two clusters based on daily summaries of time-at-depth and time-at-temperature data. Clusters reflect the C) general seasonal latitudinal distribution of basking sharks occupying shelf habitats during summer and migrating offshore during winter. Grey points in panel A) indicate that no TAD and/or TAT data were available for that day, and thus, there is no cluster assignment. The black line in panel A) indicates the climatological position of the northern edge of the Gulf Stream. The heatmap in panel B) indicates the proportion of time spent in each depth (top) or temperature (bottom) bin during each day of data. The black triangles in panel C) represent the beginning of migration calculated as encountering at least 15ºC at > 200 m depth from 16 tags with high-resolution time series data. Note the irregular depth bin intervals on the y-axis in panel **b.** World map data was obtained from Natural Earth and bathymetry data from NOAA National Centers for Environmental Information.

Of the 57 tagged individuals, 37 yielded deployments longer than 50 days. The resulting 8345 days of data from these 37 individuals captured seasonal, basin-scale migrations from the tagging area on the Northeast US shelf to the Sargasso Sea, Caribbean and into the South Atlantic (Fig 1A). All sharks were > 4.6 m long and 87.5% (14 of 16) of sharks with estimated lengths in this study were adults based on estimated basking shark length at maturity [51]. Among sharks with known sex, most were female (6 females, 2 males); however, sex was excluded from analysis because it was unknown for 29 individuals. Daily summaries of TAD and TAT data were available for 4235 deployment days spread throughout these large-scale movements. Transmitted time series data were also available for 16 individuals representing 2252 days with a temporal resolution of 75, 300, or 600 seconds and two recovered tags with resolutions of 3 seconds (Shark 1) and 30 seconds (Shark 2) spanning 213 total days. The timing of the start of migration away from the Northeast US shelf was variable, with one individual migrating as early as August 30 and one starting the migration as late as January 19. Migrations were most often initiated in October (6 of 16 individuals with high-resolution time series data).

**Fig 2:**
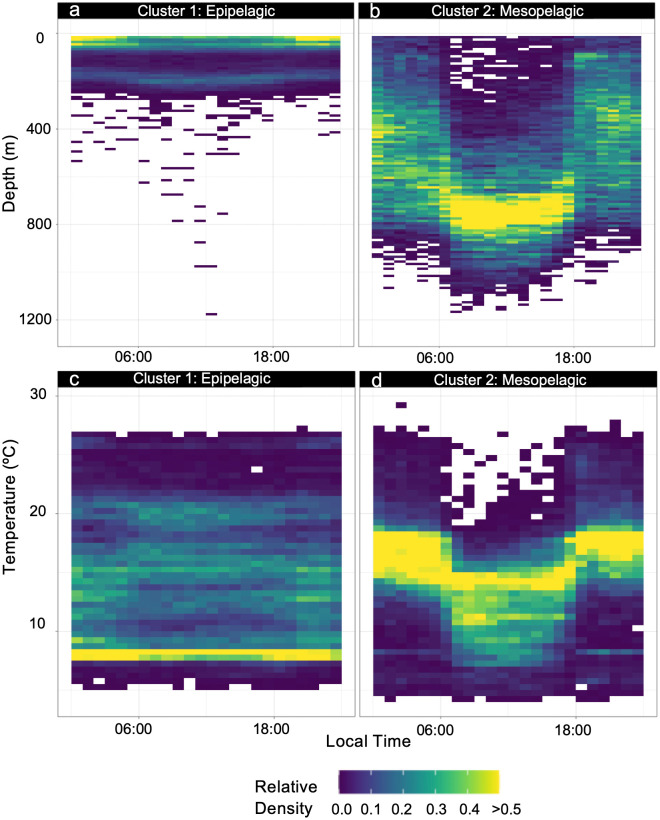
Diel depth and temperature use. Diel depth use for cluster 1 (A) and cluster 2 (B) and diel temperature use for cluster 1 (C) and cluster 2 (D) created from time series data of 16 tags.

The majority of clustering indices indicated that two clusters optimally described the variability within the basking shark TAD and TAT data (Fig 1B). This variability reflected the marked shift from occupation of shallow, epipelagic waters while on the continental shelf to the mesopelagic zone once the sharks crossed the Gulf Stream (Fig 1A, 1C). Cluster 1 included 2129 days (50.3% of data) of variable depth use concentrated primarily within the epipelagic zone (0-200m, Fig 1B, 2A). Most days within this ‘Epipelagic’ cluster occurred on the continental shelf, as far south as Virginia and as far north as Nova Scotia, or near the shelf within the Slope Sea (Fig 1A). Of the 2129 days assigned to Cluster 1 using the TAD data, higher resolution time series data were available for 1011 days (47.5% of Cluster 1 days), which indicated sharks primarily occupied the top 50 m of the water column with a secondary mode at ~200m (Fig 2A). This pattern was evident throughout all times of day (Fig 2A). Sharks experienced a broad temperature range within Cluster 1 (5–28°C; mean = 10°C), with minimal diel variability during occupancy of cooler shelf waters (Fig 2C).

Cluster 2 included 2106 days (49.7% of data) that were characterized by occupation of the mesopelagic, with the highest concentration of time spent between 400-1000m (Fig 1B, 2B). The daily, across-individual mean indicated that basking sharks spent on average 71.3% of each day in this depth range. This ‘Mesopelagic’ cluster occurred in pelagic/offshore environments, beginning at the north wall of the Gulf Stream (~40 ºN) and extending through the Sargasso Sea to the farthest southerly extent of the recorded movements (~10ºS; Fig 1A). Of the 2106 days assigned to Cluster 2 using the combined TAD and TAT data, higher resolution time series data were available for 915 days (43.4% of Cluster 2 days) which indicated sharks used much of the upper mesopelagic at night (broad use of 200-800m), while vertical habitat use was more focused during the day within the ~ 650-850m range (Fig 2B). In Cluster 2, sharks occupied generally warmer waters (mean: 15ºC, range: 4–30ºC) with minimal diel variability and exhibited pronounced diel temperature variability associated with vertical migration (Fig 2D).

**Fig 3:**
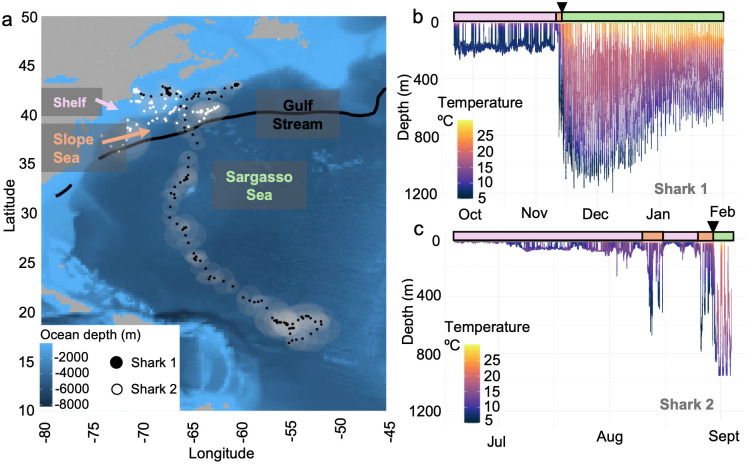
Horizontal and vertical space use by basking sharks. **A)** The estimated tracks of two basking sharks whose tags were recovered, providing the high-resolution depth temperature time series on the right **(B, C)**. Black dots represent the track that corresponds to the time series for Shark 1 (B) and white dots represent the track that corresponds to the time series for Shark 2 **(C)**. Gray areas surrounding points indicate location error. In panel A, colored text labels indicate regions of the NWA. The corresponding color bars above panels B and C indicate when the sharks spent time within these regions. Triangles above panels B and C represent the start of migration for each individual. World map data was obtained from Natural Earth and bathymetry data from NOAA National Centers for Environmental Information.

**Fig 4:**
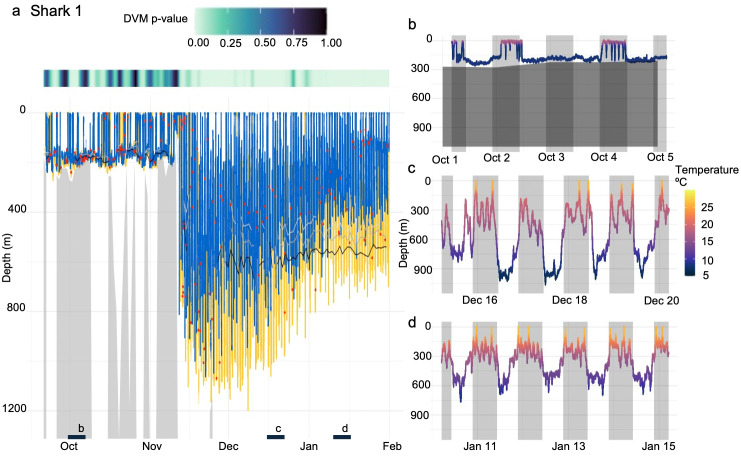
Depth time series and DVM patterns for Shark 1. **(A)** Time series depth data from the deployment of Shark 1. Yellow lines represent daytime movements, and blue represents nighttime movements. Isolumes are represented by light grey (LL50), grey (LL40), and black (LL30) lines. Red points indicate detected bioluminescence. The gray shaded areas beneath the time series represent bathymetry. The significance of DVM patterns is displayed above panel **A.** Panels B-D represent 5-day sections of data from within the tag deployment that are representative of different modes of vertical habitat use during the night (grey shading) and day (no shading). Panel B also displays bottom depth (dark grey horizontal shading).

**Fig 5:**
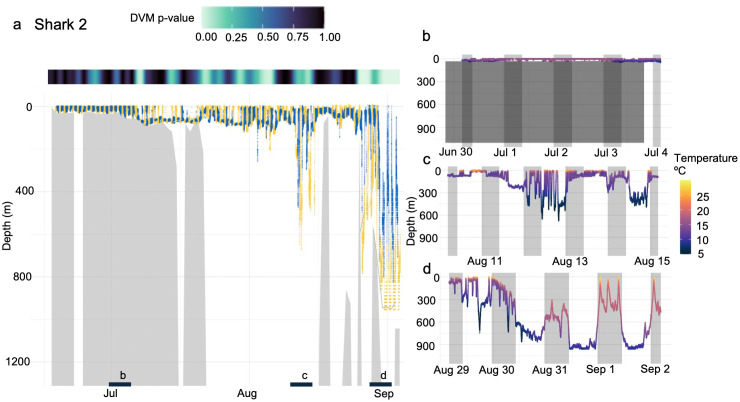
Depth time series and DVM patterns for Shark 2. **(A)** Time series depth data from the deployment of Shark 2. Yellow lines represent daytime movements, and blue represents nighttime movements. Isolumes are represented by light grey (LL50) and grey (LL40) lines. The gray shaded areas beneath the shark time series movements represent bathymetry. The significance of DVM patterns is displayed above panel **A.** Panels B-D represent 5-day sections of data from within the tag deployment that are representative of different modes of vertical habitat use during the night (grey vertical shading) and day (no shading). Panel B also displays bottom depth (dark grey horizontal shading). Note that the coarse temporal resolution of this archival dataset (30s) prohibited bioluminescence detection.

The two recovered tags provided an opportunity for more detailed analysis of the potential drivers of the observed variability in vertical habitat during each migration (Fig 3). The high-resolution, depth-temperature data for Shark 1 (Fig 3B) highlighted the contrasting vertical habitat use across oceanographic regimes that was characteristic of the broader pattern observed across all individuals: significant time near the seafloor in shelf waters ~ 200m depth and a rapid transition to mesopelagic occupation in the warmer Sargasso Sea. In addition to the shelf vs Sargasso contrast in vertical habitat use, Shark 2 also occupied the Slope Sea for several weeks in early Fall which was typified by deep excursions to nearly 800 m despite a strong water column thermal gradient and cold temperatures at depth (~5ºC at 800m; Fig 3C). A wavelet analysis of the depth-time series of these two archival tags revealed that both sharks exhibited strong, persistent DVM off the shelf, whereas significant diel depth-use patterns on the continental shelf were detected but less persistent. For Cluster 1, a significant 24-h period was detected on 25.5% of days for Shark 1 and 7.5% for Shark 2 (Fig 4–5). In contrast, a 24-h periodicity occurred on most Cluster 2 days (90.9% and 69.2% for Sharks 1 and 2, respectively; p < 0.05), indicating a strong and consistent offshore DVM signal (Fig 3–5). Shark 2 exhibited limited DVM behavior during its deployment. This likely reflects its predominantly shelf- and Slope Sea-based residency (66 of 79 days), with only 13 days spent beyond the Gulf Stream where DVM was most pronounced in other individuals.

Light levels at depth recorded by both tags followed expected biome-level patterns in water column light attenuation (Fig 4, 5), with data from Shark 1 indicating that LL30 occurred at ~200m in shelf waters, on average, and deepened to ~600m in offshore waters (Fig 4, 5). The higher-resolution light archive recorded by Shark 1 (3-sec) indicated this individual encountered bioluminescent organisms (174 detections) throughout the water column (Fig 4A). While the shark was on and near the continental shelf, detections occurred mainly in deeper waters (150-250m), presumably near the seafloor, with a few events closer to the surface. During this time, mean number of detections per day was 4 (min = 1, max = 10). While in pelagic waters off the continental shelf, these bioluminescent events occurred almost exclusively in the mesopelagic, including within the depth ranges of well-documented scattering layers. Here, the mean number of bioluminescent detections per day was 2.2 (min = 1, max = 6). Across the entire deployment, 66 bioluminescent detections (38%) occurred during the daytime compared to 108 detections (62%) at night. This presence-only detection method relies on rapid increase in tag-measured light orders of magnitude above ambient, therefore biasing detections toward low light conditions.

## Discussion

Our analysis of time-at-depth and -temperature patterns from PSAT-tagged basking sharks revealed two distinct modes of vertical habitat use that correspond to major environmental transitions during seasonal migrations. On the continental shelf, sharks were constrained by bottom depth (~200 m) and regularly moved between the surface and seafloor. In contrast, offshore movements were characterized by consistent, often prolonged, mesopelagic occupancy and strong DVM patterns. These contrasting vertical modes reflect seasonal redistribution across oceanographic regimes and provide a framework for interpreting how basking sharks use both shelf and open-ocean habitats.

### Vertical habitat use in contrasting oceanographic regimes

Behaviors within the ‘Epipelagic’ cluster occurred primarily on or near the Northeast US continental shelf during summer. Shelf waters in this region support predictable seasonal peaks in copepod abundance (e.g., *Calanus* spp.) and host a suite of planktivores that track these resources [5,52–54]. Despite being well-documented surface feeders in shelf systems [21], basking sharks in this study also used near-bottom waters, consistent with vertically compressed prey fields generated by shallow bathymetry [55]. Previous autonomous vehicle observations in Scottish shelf systems similarly documented basking sharks spending considerable time within a few meters of the seabed, although no feeding was observed during those short tracking periods [56], which mirrors results from recent satellite tag-based results from this region [44]. When zooplankton vertical migration is constrained by bottom depth, dense aggregations can form near the seafloor, a mechanism also exploited by North Atlantic right whales [57]. Together, these observations indicate that shelf-associated vertical behavior is consistent with exploitation of seasonally concentrated zooplankton resources. However, behavior associated with the epipelagic cluster was present year-round (Fig 1C). Just a few (2−4) individuals remained at high latitudes rather than migrating south during winter/early spring. This behavior suggests that, while most sharks track seasonal productivity and seemingly more hospitable habitats, they can overwinter in cold shelf environments. Basking sharks exhibit regional endothermy [58], which may facilitate tolerance of low temperatures (as low as −0.6ºC; [44]) and allow extended use of high-latitude habitats. Similar behavior has been documented in the Northeast Atlantic, where one of two individuals tagged off the coast of Norway overwintered in the Barents Sea [44] and at least six of 28 individuals tagged off the United Kingdom overwintered within proximal waters [25]. Such variability is also observed in other large pelagic fishes, in which some individuals migrate to distinct overwintering grounds (e.g., common thresher shark [59]) or forego a seasonal migration altogether (e.g., albacore tuna [60]), likely mediated by elevated thermal capacity. In this context, the year-round presence of the epipelagic cluster likely reflects individual variation in movement strategy rather than a breakdown of the broader pattern of seasonal prey tracking.

Once sharks crossed the Gulf Stream, vertical behavior shifted markedly. The ‘Mesopelagic’ cluster was defined by persistent use of 400–1000 m depths and strong DVM (Fig 2). Offshore depth distributions overlapped with depth strata associated with potential mesopelagic prey layers in the Sargasso Sea and adjacent regions [15,61,62]. This shift reflects a transition from spatially constrained shelf foraging to overlap with vertically structured prey fields in oligotrophic offshore waters.

Depth use also varied seasonally within the offshore cluster. Early in migration, Shark 1 occupied depths associated with the deeper, weakly migrating secondary DSL before shifting toward shallower depths later in winter and spring. Previous research indicates peak calanoid copepod abundance in the upper ~500 m of the Sargasso Sea during spring, consistent with the shallower dive behavior observed during that period [61]. Seasonal analyses of backscatter in the North Atlantic similarly show peak optical scattering from mesopelagic organisms is deepest in winter and shoals in spring [63]. However, spatial heterogeneity in mesopelagic layer depth across the basin is also pronounced, suggesting that the observed depth transition could reflect both seasonal vertical migration and spatial movement across distinct hydrographic regimes [63,64]. The seasonal transition in basking shark vertical habitat use in this study suggests flexible use of mesopelagic prey layers across changing oceanographic conditions and dynamic prey resources, and potential tracking of different layers over time.

High-resolution light data from Shark 1 provide independent, presence-only evidence of organismal encounter at mesopelagic depths and into the upper bathypelagic. Numerous short-duration bioluminescent flashes were recorded, including events at depths exceeding 1000 m. Mesopelagic fishes (e.g., myctophids and bristlemouths), cephalopods, and many crustaceans (including copepods) exhibit bioluminescence [65,66], and such signals are common within scattering layers. Although detection probability varies with ambient light conditions and these data do not quantify prey density, they confirm that sharks occupied depth ranges inhabited by bioluminescent mesopelagic organisms and were within ~ 3m of these taxa (i.e., the maximum detection distance of the tag’s light sensor [50]). Importantly, mesopelagic occupancy was observed across individuals through clustering analysis, independent of bioluminescence detections from the two recovered tags.

### A proposed ecological link to the secondary deep scattering layer

A consistent feature of the offshore records was repeated use of depths associated with the secondary DSL in the southern Sargasso Sea (~800–900 m), as evidenced in the time series data in Cluster 2 (Fig 2B) and the high-resolution archival data for Sharks 1 and 2 (Fig 4, 5). Scattering layers in this region are vertically structured and taxonomically diverse [15,18,62]. The primary DSL typically occupies ~400–700 m during the day and contains small mesopelagic fishes such as myctophids, along with crustaceans and zooplankton [15,61]. In contrast, deeper and more weakly migrating or non-migratory layers near ~800–900 m are commonly associated with bristlemouths (e.g., *Cyclothone*) and other small mesopelagic and bathypelagic taxa, such as hatchetfishes and dragonfishes [15,62]. Acoustic classification and net sampling indicate that these deeper layers comprise mixed assemblages of small fishes, crustaceans, gelatinous organisms, and other “fluid-like” scatterers, with relative contributions varying regionally and seasonally [14,15,17,18,62].

Small-bodied fishes such as bristlemouths are abundant within deep, weakly migrating scattering layers. The family’s most prolific genus, *Cyclothone*, is believed to be the most abundant vertebrate on Earth [67,68]. Although *Cyclothone* are not confined exclusively to the secondary DSL, they are commonly associated with this layer in the Sargasso Sea [15,69]. These fishes are small (typically ~20–70 mm), weak swimmers, and frequently captured in large numbers in midwater trawls [70–72], suggesting limited evasion capacity relative to larger, more agile mesopelagic fishes. Their body size overlaps with the size range of prey items documented in basking shark stomach contents [21], indicating that small mesopelagic fishes fall within the range that basking sharks are capable of retaining during filtration.

Repeated occupancy of depths corresponding to the secondary DSL is notable because this layer has not traditionally been considered a major foraging target for large vertebrate predators [31], most of which are pursuit feeders that target individual prey items. For most predator species, foraging on small-bodied organisms at 800–900 m imposes substantial energetic and physiological constraints, including thermal limitations in ectothermic sharks [73] and breath-hold constraints in air-breathing divers [74,75]. Thus, the energetic return on individually small prey may not offset the costs of repeated deep diving for most predators.

In contrast, basking sharks combine two traits that may relax these constraints: bulk filter feeding and physiological capacity for sustained deep occupancy. As filter feeders, energetic return depends primarily on prey density and volumetric encounter rate rather than individual prey size [76,77]. Energy intake scales with the product of prey concentration and filtration rate, meaning that even extremely small organisms can contribute substantially to net gain when encountered in sufficiently dense aggregations [78]. At the same time, basking sharks exhibit regional endothermy and substantial thermal inertia [44,58], permitting prolonged residence in deep offshore waters. Few large pelagic vertebrates combine efficient bulk filtration of small prey with the physiological capacity to remain at depth for extended periods. This combination in basking sharks may work synergistically to reduce both encounter and physiological constraints that otherwise limit access to deep, weakly migrating scattering layers for many other predator species.

Dense, multi-taxa assemblages of small-bodied mesopelagic organisms may represent energetically viable resources for basking sharks during offshore residency. The abundance, depth distribution, and limited escape capacity of *Cyclothone* illustrate how biomass concentrated in deeper scattering layers could be accessible to and profitable for a predator that combines bulk filtration with sustained deep habitat use. The repeated occupancy of these depths that we found, together with the reported composition of scattering layer communities and basking shark physiology and feeding morphology, supports a plausible trophic link between this species and biomass in the deep pelagic ocean. Direct dietary or biochemical evidence from offshore individuals will be required to resolve the extent to which mesopelagic vertebrates contribute to basking shark diet and to close this important gap in our understanding of their trophic ecology.

### Dietary Records and Offshore Foraging Potential

Nearly all diet data published on basking sharks derive from surface observations or stranded individuals collected in shelf habitats [21,79,80]. While basking sharks are generally considered zooplanktivores, stomach contents have included fish eggs, pelagic shrimp, and other small crustaceans [21,79]. No confirmed records document consumption of mesopelagic fishes, but this absence likely reflects strong geographic sampling bias toward coastal systems. The only documented deep-water prey ingestion involved pelagic shrimp (*Sergestes similis*) 40–54 mm in length consumed at>100m depth [21,79], demonstrating that basking sharks are capable of locating, capturing, and ingesting relatively large, mobile prey items at depth. These shrimp are generally considered macroplanktonic and are known to form dense aggregations, suggesting that such feeding may occur within concentrated prey fields. Regardless, because dietary data are almost exclusively derived from shelf-associated individuals, the contribution of offshore mesopelagic prey to overwintering foraging ecology remains unresolved.

### Limitations and future work

Although this study provides evidence of overlap with mesopelagic prey resources, several limitations preclude a more definitive interpretation of basking shark foraging ecology. Concurrent hydroacoustic data on scattering layer structure and composition were not available during tag deployments and were therefore inferred from published observations. Previous work has attempted basin-scale alignment of predator dive data with modeled DSL depth [31], but model-derived estimates of the primary DSL vary substantially across regions and seasons and do not provide any inference on secondary DSLs. Accordingly, our inferences rely on clustering of vertical habitat use, regional descriptions of scattering layer structure, and *in situ* proxies such as isolume depth and bioluminescence detection. Future research integrating multi-sensor biologgers, animal-borne cameras, and surface or *in situ* acoustic surveys (e.g., [81,82]) would substantially improve understanding of prey community composition and distribution and illuminate predator–prey interactions at depth.

## Conclusion

The distinct seasonal modes of vertical habitat use documented here demonstrate that basking sharks occupy shelf-associated epipelagic habitats during peak productivity and offshore mesopelagic strata during long-distance migrations. This pattern is consistent with a shift from seasonally concentrated zooplankton resources on the continental shelf [83] to vertically structured mesopelagic prey fields in oligotrophic waters. Repeated occupancy of depths associated with the secondary DSL suggests that basking sharks may access biomass near the mesopelagic–bathypelagic boundary, facilitated by the rare combination of bulk filter-feeding morphology and physiological capacity for sustained deep residence. These findings suggest that predictable mesopelagic prey resources may structure the migration phenology of this endangered species at basin scales (sensu [27]). As anthropogenic pressures on the deep ocean expand, recognizing the contribution of mesopelagic food webs to the life history of marine megafauna becomes essential [84,85]. Basking sharks may represent a key example of how energy is integrated across surface and deep-pelagic habitats, and how future ‌‌changes to midwater ecosystems [86] could reverberate through upper trophic levels.

## References

[1] Block BA, Jonsen ID, Jorgensen SJ, Winship AJ, Shaffer SA, Bograd SJ, et al. Tracking apex marine predator movements in a dynamic ocean. Nature. 2011;475(7354):86–90. doi: 10.1038/nature10082 21697831

[2] Abrahms B, Hazen EL, Aikens EO, Savoca MS, Goldbogen JA, Bograd SJ. Memory and resource tracking drive blue whale migrations. Proceedings of the National Academy of Sciences. 2019;116(12):5582–7. doi: 10.1073/pnas.1819031116 PMC6431148 30804188

[3] Del Raye G, Jorgensen SJ, Krumhansl K, Ezcurra JM, Block BA. Travelling light: white sharks (Carcharodon carcharias) rely on body lipid stores to power ocean-basin scale migration. Proc Biol Sci. 2013;280(1766):20130836. doi: 10.1098/rspb.2013.0836 23864595 PMC3730586

[4] Whitt A, Dudzinski K, Laliberté J. North Atlantic right whale distribution and seasonal occurrence in nearshore waters off New Jersey, USA, and implications for management. Endang Species Res. 2013;20(1):59–69. doi: 10.3354/esr00486

[5] Lesage V, Gavrilchuk K, Andrews R, Sears R. Foraging areas, migratory movements and winter destinations of blue whales from the western North Atlantic. Endang Species Res. 2017;34:27–43. doi: 10.3354/esr00838

[6] Wakefield ED, Miller DL, Bond SL, Le Bouard F, Carvalho PC, Catry P. The summer distribution, habitat associations and abundance of seabirds in the sub-polar frontal zone of the Northwest Atlantic. Prog Oceanogr. 2021;198:102657. doi: 10.1016/j.pocean.2021.102657

[7] Campana SE, Dorey A, Fowler M, Joyce W, Wang Z, Wright D, et al. Migration pathways, behavioural thermoregulation and overwintering grounds of blue sharks in the Northwest Atlantic. PLoS One. 2011;6(2):e16854. doi: 10.1371/journal.pone.0016854 21373198 PMC3044145

[8] Skomal G, Braun C, Chisholm J, Thorrold S. Movements of the white shark Carcharodon carcharias in the North Atlantic Ocean. Mar Ecol Prog Ser. 2017;580:1–16. doi: 10.3354/meps12306

[9] Skomal G, Marshall H, Galuardi B, Natanson L, Braun CD, Bernal D. Horizontal and vertical movement patterns and habitat use of juvenile porbeagles (Lamna nasus) in the western North Atlantic. Frontiers in Marine Science. 2021;8:624158. doi: 10.3389/fmars.2021.624158

[10] Pate JH, Wilmott JR, Jones C, Horn C, Farmer NA. Multiple datasets confirm range extension of the sicklefin devil ray Mobula tarapacana in the western North Atlantic Ocean off the eastern USA. J Mar Biol Assoc U K. 2023;103:e30. doi: 10.1017/S002531542300022X

[11] Arostegui MC, Mears D, Gaube P, Braun CD. Movement ecology of a deep-pelagic mesopredator, the bigscale pomfret: implications for pelagic food web connectivity and fishery susceptibility. Mar Ecol Prog Ser. 2025;769:183–96. doi: 10.3354/meps14934

[12] Benoit-Bird KJ, Lawson GL. Ecological insights from pelagic habitats acquired using active acoustic techniques. Ann Rev Mar Sci. 2016;8:463–90. doi: 10.1146/annurev-marine-122414-034001 26515810

[13] Cotter E, Bassett C, Lavery A. Classification of broadband target spectra in the mesopelagic using physics-informed machine learning. J Acoust Soc Am. 2021;149(6):3889–901. doi: 10.1121/10.0005114 34241451

[14] Proud R, Handegard NO, Kloser RJ, Cox MJ, Brierley AS. From siphonophores to deep scattering layers: uncertainty ranges for the estimation of global mesopelagic fish biomass. ICES J Mar Sci. 2019;76(3):718–33. doi: 10.1093/icesjms/fsy037

[15] Marohn L, Schaber M, Freese M, Pohlmann JD, Wysujack K, Czudaj S, et al. Distribution and diel vertical migration of mesopelagic fishes in the Southern Sargasso Sea — observations through hydroacoustics and stratified catches. Mar Biodivers. 2021;51(6):87. doi: 10.1007/s12526-021-01216-6

[16] Irigoien X, Klevjer TA, Røstad A, Martinez U, Boyra G, Acuña JL, et al. Large mesopelagic fishes biomass and trophic efficiency in the open ocean. Nat Commun. 2014;5:3271. doi: 10.1038/ncomms4271 24509953 PMC3926006

[17] Ariza A, Landeira JM, Escánez A, Wienerroither R, Aguilar de Soto N, Røstad A, et al. Vertical distribution, composition and migratory patterns of acoustic scattering layers in the Canary Islands. Journal of Marine Systems. 2016;157:82–91. doi: 10.1016/j.jmarsys.2016.01.004

[18] D’Elia M, Warren JD, Rodriguez-Pinto I, Sutton TT, Cook A, Boswell KM. Diel variation in the vertical distribution of deep-water scattering layers in the Gulf of Mexico. Deep Sea Research Part I: Oceanographic Research Papers. 2016;115:91–102. doi: 10.1016/j.dsr.2016.05.014

[19] Robinson C, Steinberg DK, Anderson TR, Arístegui J, Carlson CA, Frost JR, et al. Mesopelagic zone ecology and biogeochemistry – a synthesis. Deep Sea Research Part II: Topical Studies in Oceanography. 2010;57(16):1504–18. doi: 10.1016/j.dsr2.2010.02.018

[20] Braun CD, Arostegui MC, Thorrold SR, Papastamatiou YP, Gaube P, Fontes J, et al. The functional and ecological significance of deep diving by large marine predators. Annual Review of Marine Science. 2022;14(1):129–59. doi: 10.1146/annurev-marine-032521-103517 34416123

[21] Sims DW. Sieving a living. Advances in Marine Biology. Elsevier. 2008. p. 171–220. doi: 10.1016/S0065-2881(08)00003-5 18929065

[22] Gore MA, Rowat D, Hall J, Gell FR, Ormond RF. Transatlantic migration and deep mid-ocean diving by basking shark. Biol Lett. 2008;4(4):395–8. doi: 10.1098/rsbl.2008.0147 18511407 PMC2610142

[23] Johnston EM, Mayo PA, Mensink PJ, Savetsky E, Houghton JDR. Serendipitous re-sighting of a basking shark Cetorhinus maximus reveals inter-annual connectivity between American and European coastal hotspots. J Fish Biol. 2019;95(6):1530–4. doi: 10.1111/jfb.14163 31621067

[24] Skomal GB, Zeeman SI, Chisholm JH, Summers EL, Walsh HJ, McMahon KW, et al. Transequatorial migrations by basking sharks in the western Atlantic Ocean. Curr Biol. 2009;19(12):1019–22. doi: 10.1016/j.cub.2009.04.019 19427211

[25] Doherty PD, Baxter JM, Gell FR, Godley BJ, Graham RT, Hall G, et al. Long-term satellite tracking reveals variable seasonal migration strategies of basking sharks in the north-east Atlantic. Sci Rep. 2017;7(1):42837. doi: 10.1038/srep42837 28216646 PMC5316944

[26] Braun CD, Skomal GB, Thorrold SR. Integrating archival tag data and a high-resolution oceanographic model to estimate basking shark (Cetorhinus maximus) movements in the western Atlantic. Frontiers in Marine Science. 2018;5:25. doi: 10.3389/fmars.2018.00025

[27] Arostegui MC, Muhling B, Culhane E, Dewar H, Koch SS, Braun CD. A shallow scattering layer structures the energy seascape of an open ocean predator. Sci Adv. 2023;9(40):eadi8200. doi: 10.1126/sciadv.adi8200 PMC10550225 37792940

[28] Howey LA, Tolentino ER, Papastamatiou YP, Brooks EJ, Abercrombie DL, Watanabe YY, et al. Into the deep: the functionality of mesopelagic excursions by an oceanic apex predator. Ecol Evol. 2016;6(15):5290–304. doi: 10.1002/ece3.2260 27551383 PMC4984504

[29] Schaber M, Gastauer S, Cisewski B, Hielscher N, Janke M, Peña M, et al. Extensive oceanic mesopelagic habitat use of a migratory continental shark species. Sci Rep. 2022;12(1):2047. doi: 10.1038/s41598-022-05989-z 35132104 PMC8821621

[30] Andrzejaczek S, Lucas TCD, Goodman MC, Hussey NE, Armstrong AJ, Carlisle A. Diving into the vertical dimension of elasmobranch movement ecology. Sci Adv. 2022;8(33):eabo1754. doi: 10.1126/sciadv.abo1754 PMC9390984 35984887

[31] Braun CD, Della Penna A, Arostegui MC, Afonso P, Berumen ML, Block BA, et al. Proceedings of the National Academy of Sciences. 2023;120(47):e2306357120. doi: 10.1073/pnas.2306357120 PMC10666118 38150462

[32] Andrzejaczek S, Gleiss AC, Pattiaratchi CB, Meekan MG. Patterns and drivers of vertical movements of the large fishes of the epipelagic. Rev Fish Biol Fisheries. 2019;29(2):335–54. doi: 10.1007/s11160-019-09555-1

[33] Curtis TH, Zeeman SI, Summers EL, Cadrin SX, Skomal GB. Eyes in the sky: linking satellite oceanography and biotelemetry to explore habitat selection by basking sharks. Anim Biotelem. 2014;2(1):12. doi: 10.1186/2050-3385-2-12

[34] Gore M, Abels L, Wasik S, Saddler L, Ormond R. Are close-following and breaching behaviours by basking sharks at aggregation sites related to courtship?. Journal of the Marine Biological Association of the United Kingdom. 2019;99(3):681–93. doi: 10.1017/S0025315418000383

[35] Sims DW, Quayle VA. Selective foraging behaviour of basking sharks on zooplankton in a small-scale front. Nature. 1998;393(6684):460–4. doi: 10.1038/30959

[36] Sims DW. Threshold foraging behaviour of basking sharks on zooplankton: life on an energetic knife-edge?. Proc R Soc Lond B. 1999;266(1427):1437–43. doi: 10.1098/rspb.1999.0798

[37] Sims DW, Witt MJ, Richardson AJ, Southall EJ, Metcalfe JD. Encounter success of free-ranging marine predator movements across a dynamic prey landscape. Proc Biol Sci. 2006;273(1591):1195–201. doi: 10.1098/rspb.2005.3444 16720391 PMC1560279

[38] R Core Team. R: A Language and Environment for Statistical Computing. https://www.R-project.org/ 2024.

[39] Tan PN, Steinbach M, Karpatne A, Kumar V. Introduction to data mining. 2nd ed. Pearson. 2019.

[40] Murtagh F, Legendre P. Ward’s hierarchical agglomerative clustering method: which algorithms implement ward’s criterion?. J Classif. 2014;31(3):274–95. doi: 10.1007/s00357-014-9161-z

[41] Charrad M, Ghazzali N, Boiteau V, Niknafs A. NbClust: An R package for determining the relevant number of clusters in a data Set. J Stat Softw. 2014;61(6). doi: 10.18637/jss.v061.i06

[42] Braun CD, Galuardi B, Thorrold SR. HMMoce: An R package for improved geolocation of archival‐tagged fishes using a hidden Markov method. Methods Ecol Evol. 2018;9(5):1212–20. doi: 10.1111/2041-210x.12959

[43] Gaube P, Braun CD, Lawson GL, McGillicuddy DJ, Penna AD, Skomal GB. Mesoscale eddies influence the movements of mature female white sharks in the Gulf Stream and Sargasso Sea. Scientific Reports. 2018;8(1):1. doi: 10.1038/s41598-018-25565-8 29743492 PMC5943458

[44] Klöcker CA, Bjelland O, Ferter K, Arostegui MC, Braun CD, da Costa I, et al. Basking sharks of the Arctic Circle: year-long, high-resolution tracking data reveal wide thermal range and prey-driven vertical movements across habitats. Anim Biotelemetry. 2025;13(1). doi: 10.1186/s40317-025-00407-3

[45] Roesch A, Schmidbauer H. WaveletComp: Computational Wavelet Analysis. 2018.

[46] Aksnes DL, Røstad A, Kaartvedt S, Martinez U, Duarte CM, Irigoien X. Light penetration structures the deep acoustic scattering layers in the global ocean. Sci Adv. 2017;3(5):e1602468. doi: 10.1126/sciadv.1602468 PMC5451191 28580419

[47] Bianchi D, Stock C, Galbraith ED, Sarmiento JL. Diel vertical migration: ecological controls and impacts on the biological pump in a one‐dimensional ocean model. Global Biogeochemical Cycles. 2013;27(2):478–91. doi: 10.1002/gbc.20031

[48] Aumont O, Maury O, Lefort S, Bopp L. Evaluating the potential impacts of the diurnal vertical migration by marine organisms on marine biogeochemistry. Global Biogeochemical Cycles. 2018;32(11):1622–43. doi: 10.1029/2018gb005886

[49] Omand MM, Steinberg DK, Stamieszkin K. Cloud shadows drive vertical migrations of deep-dwelling marine life. Proc Natl Acad Sci. 2021;118(32):e2022977118. doi: 10.1073/pnas.2022977118 PMC8364114 34349017

[50] Vacquié-Garcia J, Mallefet J, Bailleul F, Picard B, Guinet C. Marine Bioluminescence: measurement by a classical light sensor and related foraging behavior of a deep diving predator. Photochem Photobiol. 2017;93(5):1312–9. doi: 10.1111/php.12776 28425091

[51] Ahnelt H, Sauberer M, Ramler D, Koch L, Pogoreutz C. Negative allometric growth during ontogeny in the large pelagic filter-feeding basking shark. Zoomorphology. 2019;139(1):71–83. doi: 10.1007/s00435-019-00464-2

[52] Mayo CA, Marx MK. Surface foraging behaviour of the North Atlantic right whale, Eubalaena glacialis, and associated zooplankton characteristics. Can J Zool. 1990;68(10). doi: 10.1139/z90-308

[53] Durbin EG, Gilman SL, Campbell RG, Durbin AG. Abundance, biomass, vertical migration and estimated development rate of the copepod Calanus finmarchicus in the southern Gulf of Maine during late spring. Continental Shelf Research. 1995;15(4–5):571–91. doi: 10.1016/0278-4343(94)00060-z

[54] Ross C, Runge J, Roberts J, Brady D, Tupper B, Record N. Estimating North Atlantic right whale prey based on Calanus finmarchicus thresholds. Mar Ecol Prog Ser. 2023;703:1–16. doi: 10.3354/meps14204

[55] Aarflot J, Dalpadado P, Fiksen Ø. Foraging success in planktivorous fish increases with topographic blockage of prey distributions. Mar Ecol Prog Ser. 2020;644:129–42. doi: 10.3354/meps13343

[56] Hawkes LA, Exeter O, Henderson SM, Kerry C, Kukulya A, Rudd J, et al. Autonomous underwater videography and tracking of basking sharks. Anim Biotelemetry. 2020;8(1). doi: 10.1186/s40317-020-00216-w

[57] Baumgartner MF, Wenzel FW, Lysiak NSJ, Patrician MR. North Atlantic right whale foraging ecology and its role in human-caused mortality. Mar Ecol Prog Ser. 2017;581:165–81.

[58] Klöcker CA, Schlindwein A, Arostegui MC, Bruvold IM, Wernström JV, Martin‐Armas M. Giants in the cold: Morphological evidence for vascular heat retention in the viscera but not the skeletal muscle of the basking shark (Cetorhinus maximus). J Fish Biol. 2025. doi: 10.1111/jfb.70052 40361299

[59] Kneebone J, Arostegui MC, Natanson LJ, Skomal GB, Braun CD, Bernal D. Horizontal and vertical movements and habitat use of the common thresher shark Alopias vulpinus in the western North Atlantic. Mar Ecol Prog Ser. 2025;773:95–113. doi: 10.3354/meps14978

[60] Muhling BA, Snyder S, Hazen EL, Whitlock RE, Dewar H, Park JY. Risk and Reward in Foraging Migrations of North Pacific Albacore Determined From Estimates of Energy Intake and Movement Costs. Frontiers in Marine Science. 2022;9:730428. doi: 10.3389/fmars.2022.730428

[61] Deevey GB, Brooks AL. Copepods of the Sargasso Sea off Bermuda: Species composition, and vertical and seasonal distribution between the surface and 2000 M. Bull Mar Sci. 1977;27(36):256–91.

[62] Proud R, Cox MJ, Guen CL, Brierley AS. Fine-scale depth structure of pelagic communities throughout the global ocean based on acoustic sound scattering layers. Mar Ecol Prog Ser. 2018;598:35–48. doi: 10.3354/meps12612

[63] Li JH, Yang J, Chen G. Temporal and spatial influences of environmental factors on the distribution of mesopelagic organisms in the North Atlantic Ocean. Biogeosciences. 2025;22(20):5635–50. doi: 10.5194/bg-22-5635-2025

[64] Allcock AL, Amon DJ, Bridges AEH, Colaço A, Escobar-Briones E, Hilário A. Deep-sea ecosystems of the North Atlantic Ocean: discovery, status, function and future challenges. Deep Sea Res Part Oceanogr Res Pap. 2025;226:104580. doi: 10.1016/j.dsr.2025.104580

[65] Johnsen S, Frank TM, Haddock SHD, Widder EA, Messing CG. Light and vision in the deep-sea benthos: I. Bioluminescence at 500-1000 m depth in the Bahamian islands. J Exp Biol. 2012;215(Pt 19):3335–43. doi: 10.1242/jeb.072009 22956246

[66] Letendre F, Twardowski M, Blackburn A, Poulin C, Latz MI. A review of mechanically stimulated bioluminescence of marine plankton and its applications. Frontiers in Marine Science. 2024;10:1299602. doi: 10.3389/fmars.2023.1299602

[67] Marshall NB. Aspects of deep sea biology. London: Hutchinson. 1954.

[68] Nelson JS, Grande TC, Wilson MVH. Fishes of the world. John Wiley & Sons. 2016.

[69] Sutton TT. Vertical ecology of the pelagic ocean: classical patterns and new perspectives. J Fish Biol. 2013;83(6):1508–27. doi: 10.1111/jfb.12263 24298949

[70] Barham EG. Deep-sea fishes: lethargy and vertical orientation. Proceedings of an International Symposium on Biological Sound Scattering in the Ocean. 1971. 100–18.

[71] Smith KL Jr, Laver MB. Respiration of the bathypelagic fish Cyclothone acclinidens. Mar Biol. 1981;61(4):261–6. doi: 10.1007/bf00401564

[72] Davison P, Lara-Lopez A, Anthony Koslow J. Mesopelagic fish biomass in the southern California current ecosystem. Deep Sea Research Part II: Topical Studies in Oceanography. 2015;112:129–42. doi: 10.1016/j.dsr2.2014.10.007

[73] Braun CD, Gaube P, Sinclair-Taylor TH, Skomal GB, Thorrold SR. Mesoscale eddies release pelagic sharks from thermal constraints to foraging in the ocean twilight zone. Proceedings of the National Academy of Sciences. 2019;116(35):17187–92. doi: 10.1073/pnas.1903067116 PMC6717292 31387979

[74] Jaud T, Dragon A-C, Garcia JV, Guinet C. Relationship between chlorophyll a concentration, light attenuation and diving depth of the Southern elephant seal Mirounga leonina. PLoS One. 2012;7(10):e47444. doi: 10.1371/journal.pone.0047444 23082166 PMC3474817

[75] Tyack PL, Johnson M, Soto NA, Sturlese A, Madsen PT. Extreme diving of beaked whales. J Exp Biol. 2006;209(21). doi: 10.1242/jeb.02505 17050839

[76] Benoit-Bird KJ. Resource patchiness as a resolution to the food paradox in the sea. Am Nat. 2024;203(1):1–13. doi: 10.1086/727473 38207143

[77] Crowder LB. Optimal foraging and feeding mode shifts in fishes. Environ Biol Fish. 1985;12(1):57–62. doi: 10.1007/bf00007710

[78] Goldbogen JA, Cade DE, Wisniewska DM, Potvin J, Segre PS, Savoca MS, et al. Why whales are big but not bigger: Physiological drivers and ecological limits in the age of ocean giants. Science. 2019;366(6471):1367–72. doi: 10.1126/science.aax9044 31831666

[79] Mutoh M, Omori M. Two records of patchy occurrences of the oceanic shrimp Sergestes similis Hansen off the east coast of Honshu, Japan. J Oceanogr. 1978;34(1):36–8. doi: 10.1007/BF02109614

[80] Matthews LH, Parker HW. Notes on the anatomy and biology of the basking shark (Cetorhinus maximus (Gunner)). Proc Zool Soc Lond. 1950;120(3):535–76. doi: 10.1111/j.1096-3642.1950.tb00663.x 24537280

[81] Yoshino K, Takahashi A, Adachi T, Costa DP, Robinson PW, Peterson SH. Acceleration-triggered animal-borne videos show a dominance of fish in the diet of female northern elephant seals. J Exp Biol. 2020. doi: 10.1242/jeb.212936 32041802

[82] Josse E, Bach P, Dagorn L. Simultaneous observations of tuna movements and their prey by sonic tracking and acoustic surveys. Advances in Invertebrates and Fish Telemetry. Springer Netherlands. 1998. p. 61–9. doi: 10.1007/978-94-011-5090-3_8

[83] Horst T, Lawton R, Toner R, Scherer M. Seasonal abundance and occurrence of some planktonic and ichthyofaunal communities in Cape Cod Bay. Observations on the ecology and biology of Western Cape Cod Bay, Massachusetts. American Geophysical Union (AGU). 1984. p. 241–61. doi: 10.1029/LN011p0241

[84] Iglesias IS, Santora JA, Fiechter J, Field JC. Mesopelagic fishes are important prey for a diversity of predators. Frontiers in Marine Science. 2023;10:1220088. doi: 10.3389/fmars.2023.1220088

[85] Willis C, Gardner KG, Arostegui MC, Braun CD, Golet W, Houghton L, et al. Evaluating the importance of mesopelagic prey to three top teleost predators in the northwest Atlantic Ocean. ICES J Mar Sci. 2025;82(3):fsaf028. doi: 10.1093/icesjms/fsaf028

[86] Ariza A, Lengaigne M, Menkes C, Lebourges-Dhaussy A, Receveur A, Gorgues T, et al. Global decline of pelagic fauna in a warmer ocean. Nat Clim Chang. 2022;12(10):928–34. doi: 10.1038/s41558-022-01479-2

